# Brain Structural Differences between Normal and Obese Adults and their Links with Lack of Perseverance, Negative Urgency, and Sensation Seeking

**DOI:** 10.1038/srep40595

**Published:** 2017-01-16

**Authors:** Haifeng Wang, Baohong Wen, Jingliang Cheng, Hongpeng Li

**Affiliations:** 1 Department of Neurosurgery, The first Bethune Hospital of Jilin University, Changchun, Jilin 130041, P.R. China; 2 Department of MRI, The First Affiliated Hospital of Zhengzhou University, Zhengzhou 450052, P.R. China; 3 Department of Radiology, The Second Hospital of Jilin University, Changchun, Jilin 130041, P.R. China

## Abstract

In order to examine the difference in brain structure between obese and normal weight individuals, and to explore the relationship between the neuroanatomical changes and impulsivity traits, this study used a voxel-based morphometry method to examine gray matter (GM) volume alterations related to impulsive personality traits in obese individuals relative to normal weight. Eighty adults that completed the UPPS-P Impulsive Behavior Scale were analyzed. Possible GM volume alterations were first analyzed at the whole brain level, and then the relationship between regional GM volume differences and UPPS-P scores were examined in selected regions of interest. Reduced GM volumes were found in the frontal and limbic regions in the obese group compared to normal weight individuals. In the normal weight group, lack of perseverance was negatively correlated with GM volume in the anterior cingulate cortex, and negative urgency was negatively correlated with GM volume in the insula. In the obese group, sensation seeking was negatively correlated with GM volume in the left amygdala and right pallidum. These findings might improve our understanding of the relationship between lack of perseverance, negative urgency, and sensation seeking and body weight fluctuations.

Obesity is a major health hazard of modern society and promotes co-morbid diseases^1^,^2^. For example, it is documented that a body mass index (BMI) of 30–35 kg/m^2^ reduces life expectancy by two to four years^3^. Overconsumption of calorie-dense foods, depression and anxiety, side effects of pharmaceuticals, or genetics all may be causal factors for obesity^4^. Impulsive personality trait is also documented to contribute to obesity^5^. Impulsivity towards food has been indicated for increased food intake in obese people, and appears more pronounced in people with binge eating disorder^2^,^6^,^7^,^8^. An impulsive personality predicts a heightened food intake and body fat in women^9^,^10^. Urgency is negatively related to self-control on eating^11^,^12^. Negative urgency is associated with food addiction directly, and this link is responsible for the relationship between food addiction and BMI^13^. Lack of perseverance is related to weight fluctuation^12^. Overweight and obese people have higher levels of urgency, lack of perseverance and sensation seeking^14^. Neuroanatomical investigations on personal impulsivity traits on the presentation of obesity may help inform the neural basis of impulsivity and ultimately benefit obesity prevention.

Neuroimaging studies reveal that impulsivity involves brain regions important in reward reinforcement and response inhibition. Brain anatomical studies have identified gray matter (GM) atrophy in impulsive individuals in the orbitofrontal cortex (OFC), anterior cingulate cortex (ACC), medial prefrontal cortex, and amygdala^15^. A study notes that a smaller OFC volume in healthy subjects relates to high impulsivity^16^. Functional neuroimaging evidence also pinpoints several brain regions corresponding to impulsiveness, including the OFC, inferior frontal gyrus, ventrolateral and dorsolateral prefrontal cortices, ACC, amygdala, ventral pallidum, insula and hippocampus^17^,^18^,^19^,^20^. In addition, pallidum activation during reward anticipation has been demonstrated to be correlated with impulsivity in alcoholics^21^.

Structural imaging studies also have uncovered lower total GM volumes and reduced regional GM volumes in the OFC of the obese relative to lean controls^11^,^12^. Moreover, Yokum *et al*. found that BMIs are correlated with volume changes in brain regions involved in reward processing and somatosensory processing^22^, whereas reduced regional GM volumes in the prefrontal cortex are correlated with higher rates of BMI increase^22^. Impulsivity plays an important role in weight gain. Maayan *et al*. reported that obese individuals are characterized by increased disinhibition and reduced cognitive control, and that both traits are correlated with reduced GM volumes in the OFC^23^. Ralph *et al*. observed an enlarged amygdala in obese subjects, which implicated the importance of the hedonic effect in the regulation of feeding^24^. These findings suggest that volumetric brain measures are useful to characterize the neurobiological underpinnings of obesity and that brain structural volumes are associated with certain disease-specific features (e.g., BMI). In the context of the current study, there is a knowledge gap in the link between brain structural alterations and impulsive traits in the presentation of adult obesity.

The objectives of this investigation are to examine the differences in brain structures between obese and normal weight individuals, and to explore the relationship between neuroanatomical changes and impulsivity traits. We hypothesize that lack of perseverance, negative urgency and sensation seeking has different links with these neuroanatomical alterations in normal weight and obese groups.

## Results

### Participants’ demographic characteristics

The demographic characteristics for the normal weight and obese groups are summarized in Table 1. The groups were matched for gender, handedness, depression status, cognitive restraint on eating and disinhibition of control. However, the obesity subjects had higher hunger scores than the normal weight group. Given that the age of the two groups was significantly different, it was used as a covariate in a further analysis.

### Trait impulsivity measures

There were no significant between group differences in lack of premeditation, lack of perseverance, sensation seeking, negative urgency and positive urgency (Table 1).

### MRI Imaging analysis

**Figure 1:**
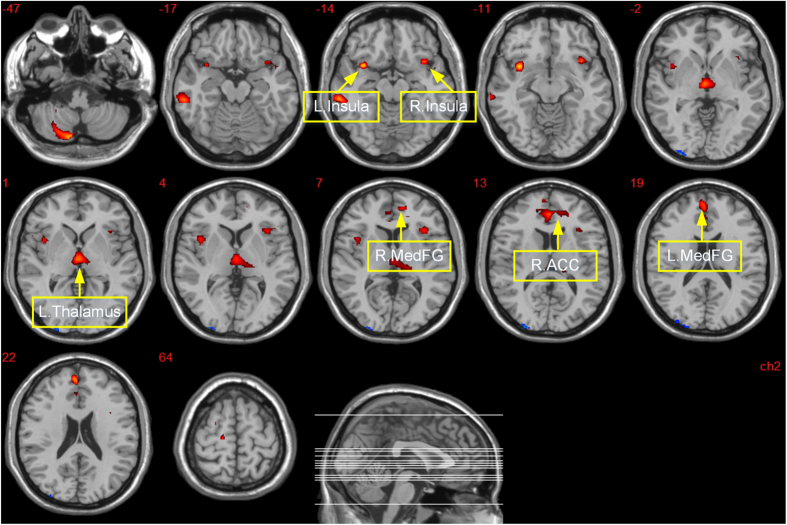
Brain mapping of GM volumes demonstrates a significant difference between the obese and normal weight group (*p* = 0.005, cluster size > 50). The red regions are reductions in GMV in the obese versus normal weight group. The blue regions are increases in GMV in the obese versus normal weight group. Abbreviations: R. MedFG = right Medial Frontal Gyrus; R. ACC = right Anterior Cingulate Cortex; L. MedFG = left Medial Frontal Gyrus.

The obese group had significantly lower GM volumes than the normal weight group in the left inferior frontal gyrus (BA 13), bilateral insula (BA 13), left pyramis, inferior semi-lunar lobule and cerebellar tonsil, bilateral medial frontal gyrus (BA 10), right anterior cingulate cortex (BA 32), bilateral thalamus and left middle frontal gyrus (BA 6) (Table 2, Fig. 1). On the other hand, the obese group showed significantly higher GM volumes in the left inferior occipital gyrus and middle occipital gyrus than in the normal weight group (Table 2, Fig. 1).

### Correlation analyses between GM and personality

Lack of perseverance was negatively correlated with GM volume in the anterior cingulate in the normal weight group (R = −0.372, *p* = 0.009) but not in the obesity group. Negative urgency was negatively correlated with GM volume within the insula in the normal weight group (R = −0.364, *p* = 0.010) but not in the obesity group. Sensation seeking was negatively correlated with GM volume in the left amygdala (R = −0.414, *p* = 0.010) and right pallidum (R = −0.448, *p* = 0.010) in the obese group.

## Discussion

In the current study, we examined the difference in brain structures between obese and normal weight individuals, and explored the relationship between neuroanatomical changes and impulsivity traits. The imaging analysis found a significant group difference in brain regions modulating impulsivity such as the ventromedial prefrontal cortex, anterior cingulate cortex, insula and thalamus. The anterior cingulate cortex and insula, which constitute the salience network, possess a significant link with a lack of perseverance and negative urgency in the normal group respectively. In addition, the left amygdala and right pallidum showed a close relationship with sensation seeking in the obese group. These results showed a different relationship between these neuroanatomical differences and impulsivity traits in normal weight and obese individuals.

In the current study, we did not find a significant group difference in cognitive restraint, disinhibition, and depression status. Some study indicated that obese subjects had poor cognitive control and high disinhibition^23^. However, another study also documented that obese subjects may attempt to curb the intake of high-calorie foods because they are aware of the weight gain effect^25^. However, in our study, there were 19 subjects’ BMIs that ranged from 30 to 35, 8 subjects’ BMIs that ranged from 35 to 40, and 4 subjects’ BMIs that ranged from 40 to 43.40. The missing significant group difference in cognitive restraint and disinhibition may be partly related to the low ratio of morbid obesity. A longitudinal study suggested that baseline obesity increased the risk of depression, and depression also promoted the odds for being overweight^26^. We need more information about the participant’s history of being obese to explain the depression and weight status. We discuss this in the limitation parts.

In line with previous studies^27^,^28^,^29^,^30^,^31^, the obese group showed decreased GM volumes in the frontal, limbic and cerebellum cortices. A previous study demonstrated that the anterior insula takes part in processing the taste, smell, texture, and fat content of foods^27^. One PET study documented that obese individuals have greater response to food tastes than lean individuals^28^. The insula, anterior cingulate cortex and medial frontal gyrus receive various homeostatic and salience information. Some studies found that the response of the ACC was negatively correlated with disinhibition, and obese individuals had less activation in the ACC than normal-weight participants^29^,^30^. GM volume of the thalamus has been suggested to be negatively associated with body fat content^31^. These findings showed decreased brain structures responding to sensory and salience processing of food in obese individuals, which were consistent with the reward-deficiency theory^32^.

In the current study, lack of perseverance was negatively correlated with GM volume in the ACC in the normal weight group. Lack of perseverance refers to failing to maintain focus on difficult or boring tasks^33^. It possesses the important relationship in decision making, for example, subjects with a low perseverance score learn more slowly in choosing from the good decks during the gambling task^34^. The ACC is involved in attention control and decision-making^29^,^35^,^36^. It plays a key role in cognitive control and reward expectation during food intake^37^,^38^. The negative association between lack of perseverance and GM volume is consistent with the items mentioned above. However, for the obese subjects, this link may be broken, suggesting that there is a change in the neural mechanism underlying cognitive control in obese people.

Negative urgency was negatively correlated with GM volume in the insula in the normal weight group but not in the obesity group. Negative urgency refers to losing control over their behavior when experiencing strong negative emotions^33^. Individuals with a high level of negative urgency had a high tendency to engage in addictive behaviors, such as consumption of alcohol and drugs^39^,^40^. Negative urgency is significantly associated with food addiction directly, and this link is also responsible for their relationship between food addiction and BMI^13^. A wealth of neuroimaging data on eating behavior indicated that difficulties in the regulation of food intake may be related to aberrant brain function in the insular cortex^41^. The insula expresses the feeling state that modulates motivational behavior in conjunction with bodily homeostasis^42^. In addition, an fMRI study found that the response in the insula underlies emotional processing in working memory^43^ and decision making. For example, its activity is related to the extent of risky decisions during the gambling task^44^. There is a negative correlation between negative urgency and GM volume in the insula in normal weight subjects, which suggests that the structure of the insula is altered in the obesity group. The insula may fail to regulate the negative emotion causing risky decisions.

Sensation seeking was negatively correlated with the GM volume in the left amygdala and right pallidum in the obese group. Sensation seeking is a personality trait to search for new experiences and feelings. It is associated with a tendency to strengthen the impact of rewards to food. Overweight and obese persons have higher levels of sensation seeking^14^. This has been positively correlated with preferences for unhealthy foods^45^. Amygdala and pallidum are implicated in the rewarding effect of food. Amygdala is involved in the conditioned response to food, and pallidum selectively responds to a cue predicting reward availability^46^. In line with the previous study, we found that GM volumes of the amygdala and pallidum were reduced in the obese group^47^, suggesting of a modulation of the alterations of the conditional response to food in obese people. The negative association between sensation seeking and GM volume of the amygdala and pallidum may further indicate that the conditional regulation is more important in obese people.

## Limitations

It is important to consider the limitations of this study. First, this study was designed to compare the GM volume alterations in obese subjects and to explore the relationship between the possible change and impulsivity traits. There is no group difference in cognitive restraint, disinhibition, and depression status, which may be partly related to the low ratio of morbid obesity and relatively small sample size. Studies with a larger body of subjects will increase the statistical power. As a cross-sectional design, the present study cannot infer causality of the relationships between GM volume alterations and impulsivity traits, which may be bi-directional and related to some latent variables. Further longitudinal studies may be required to improve the understanding of the link between GM volume alterations and impulsivity traits. Second, there is a significant group difference in age. The previous study has documented that there is an age difference in the impulsivity trait^48^. Although age is included in the analysis as a covariate, the potential influence of age should be further noted. For example, what is the relationship between BMI and impulsivity change modulated by age? We need more information to answer this question. Further studies may be required to answer this question.

## Conclusion

In the current study, we investigated the difference in brain structures between obese and normal weight individuals, and explored the relationship between the neuroanatomical changes and impulsivity traits. Compared with normal weight controls, obese subjects showed reduced GM volume in cortices responding to reward and salience encoding. The impulsivity traits showed different relationships with brain regions between obese and normal weight control patients.

## Methods

### Participants and MRI acquisition

MRI data were obtained from an open data website (http://fcon_1000.projects.nitrc.org/indi/pro/nki.html), provided by the Center for Advanced Brain Imaging of the Nathan S. Kline Institute^49^. The imaging data of eighty adults (18–55 years old) were analyzed in the current study. The participants were initially classified as normal weight adults (n = 49, mean BMI = 21.87, SE = 0.29) and obese (n = 31, mean BMI = 34.38, SE = 0.69) according to their BMI following the International Obesity Task Force (IOTF) criteria^14^,^15^. All of the subjects possessed no history of psychiatric disorders or any neurological illnesses. Informed written consent was obtained prior to the image scans. The scans were conducted in accordance with the Institutional Review Board guidelines from the Center for Advanced Brain Imaging of the Nathan S. Kline Institute and in compliance with the Declaration of Helsinki^49^.

Participants were scanned in a 3.0 T whole body MRI scanner (Siemens MAGNETOM TrioTim Syngo MR). A T1-weighted 3D volume was acquired for each participant using a T1-weighted 3D-turbo-gradient echo sequence in sagittal orientation with a 0.94 × 0.94 × 1.0 mm resolution (200 Transverse slices, FOV = 240 × 240 mm^2^, matrix 256 × 256), TR = 2500 ms, TE = 3.5 ms, TI = 1200 ms, slice thickness = 1 mm and Flip angle = 8°. The sequence was optimal for reducing field inhomogeneity, susceptibility artifacts and motion sensitivity.

### Measure of impulsivity

Impulsivity scores were assessed with a UPPS-P Impulsive Behavior Scale. The UPPS-P Impulsive Behavior Scale^33^,^50^ is a 59-item inventory. It was designed to measure five distinct personality pathways characterizing impulsive behaviors: sensation seeking, perseverance, premeditation, negative urgency and positive urgency. We used the scores of the sum of each of these five UPPS–P dimensions for the analyses^51^. In addition, depression scores were assessed with Beck Depression Inventory-II^52^. Eating behavior scores were evaluated with the Three Factor Eating Questionnaire^53^ during fasting state.

### GM Volumetric Analysis

All MPRAGE images were pre-processed and analyzed using SPM8 (http://www.fil.ion.ucl. ac.uk/spm). In order to screen for artifacts or gross anatomical abnormalities, each MR image was first displayed in SPM8. Images were reoriented manually to be set to the anterior commissure for better registration. MPRAGE images of each subject were then spatially normalized to the standard T1 Montreal Neurological Institute template and segmented into gray matter (GM), white matter (WM) and cerebrospinal fluid (CSF) using the tissue classification algorithm in SPM8. The segmented partitions were subsequently normalized to their respective standard templates. The normalized, segmented gray matter images were then modulated by calculating the Jacobian determinants derived from the special normalization step, and multiplying each voxel by the relative change in volume^54^. Finally, images were smoothed with a 3-D Gaussian filter of 8 mm^3^ full width at half maximum (FWHM) to increase the signal to noise ratio.

### ROI analysis

The anatomic region of interest (ROI) was anatomically selected based on previous evidence of its involvement in adult obesity, including the OFC, amygdala, and pallidum^18^,^20^. ROIs were created by the Wake Forest University (WFU) PickAtlas toolbox^55^.

### Statistical Analyses

Two groups of subjects, normal weight (BMI < 25) and obese (BMI > 30), were assessed for their GM volume values. The voxel-wise two-sample *t*-tests were performed to compare the group differences in GM volumes. The resulting statistical maps with a significant level of *p* = 0.005, cluster size > 50 were identified as activations^51^. The GM volume values of the significant activations and each ROI (OFC, amygdala and pallidum) were calculated for each subject. To compare structural brain alterations related to impulsive personality traits between the two groups, we used a partial correlation analysis with age, gender and handiness as covariates to analyze the relationship between regional GM volumes of ROIs and the scores of each impulsive personality traits scale (lack of premeditation, lack of perseverance, sensation seeking, negative urgency, and positive urgency). The correlation with a significance of p = 0.010 was further discussed.

## Additional Information

**How to cite this article**: Brain Structural Differences between Normal and Obese Adults and their Links with Lack of Perseverance, Negative Urgency, and Sensation Seeking. *Sci. Rep.* **7**, 40595; doi: 10.1038/srep40595 (2017).

**Publisher's note:** Springer Nature remains neutral with regard to jurisdictional claims in published maps and institutional affiliations.

## Figures and Tables

**Table 1:** Demographic and subjects characteristics.

	Normal weight (n = 49) Mean (SE)	Obesity (n = 31)	P Value
Age (years)	29.55 (1.41)	39.58 (1.93)	*t *=* −*4.283, *p* < 0.001
Gender (male/female)	28/21	24/7	*t *=* −*1.870, *p* = 0.065
Hand (left/right)	7/42	5/26	*t *=* *0.219, *p* = 0.828
BMI	21.87 (0.29)	34.38 (0.69)	*t *=* −*18.975, *p* < 0.001
Lack of premeditation^1^	20.45 (0.77)	20.26 (0.95)	*t *=* 0*.155, *p* = 0.877
Lack of perseverance^1^	18.24 (0.64)	18.42 (0.81)	*t *=* *−0.170, *p* = 0.866
Sensation seeking^1^	36.27 (1.04)	32.74 (1.71)	*t *=* *1.869, *p* = 0.065
Negative urgency^1^	20.98 (1.06	20.87 (1.32)	*t *=* 0*.064, *p* = 0.949
Positive urgency^1^	21.10 (1.17)	20.68 (1.22)	*t *=* *−0.240, *p* = 0.811
Depression^2^	4.76 (1.02)	6.03 (1.66)	*t *=* −*0.696, *p* = 0.489
Hunger^3^	3.59 (0.41)	5.45 (0.60)	*t *=* −*2.668, *p* = 0.009
Disinhibition^3^	4.16 (0.47)	4.35 (0.58)	*t *=* −*0.256, *p* = 0.799
Dietary restraint^3^	6.49 (0.64)	7.03 (0.96)	*t *=* −*0.492, *p* = 0.624

^1^Impulsivity scores were assessed with UPPS-P Impulsive Behavior Scale;

^2^Depression scores were assessed with Beck Depression Inventory-II;

^3^Eating behaviors were assessed with Three Factor Eating Questionnaire.

**Table 2:** Brain mapping of GM volumes demonstrates significant difference between obese and normal weight group (*p* = 0.005, cluster size > 50).

Region	BA	Voxel	Z	MNI		
				X	Y	Z
***Normal control vs. obese group***						
L.Insula	13	145	4.14	−36	8	−14
L.Insula	13		3.37	−42	11	1
L.Inferior Frontal Gyrus	13		2.78	−30	26	−11
L.Pyramis	Pulvinar	175	4.04	−6	−82	−47
L.Inferior Semi-Lunar Lobule	*		3.69	−24	−73	−47
L.Cerebellar Tonsil	*		2.94	−27	−37	−47
L.Inferior Temporal Gyrus	20	90	3.9	−63	−34	−17
L.Medial Frontal Gyrus	10	332	3.86	−6	53	22
R.Anterior Cingulate	32		3.64	0	41	13
R.Medial Frontal Gyrus	10		3.53	9	47	7
L.Thalamus	Pulvinar	215	3.74	−3	−13	−2
R.Thalamus	Pulvinar		3.09	18	−28	7
R.Insula	13	142	3.69	36	14	−14
R.Insula	13		3.4	36	20	4
R.Insula	13		3.05	33	11	19
L.Middle Frontal Gyrus	6	67	3.54	−15	−22	64
L.Middle Frontal Gyrus	6		3.17	−27	−1	64
***Obese group vs. normal control***						
L.Middle Occipital Gyrus	18	85	3.95	−30	−100	13
L.Inferior Occipital Gyrus	17		3.59	−27	−103	−2
L.Middle Occipital Gyrus	19		3.01	−33	−94	22
